# First case of infective endocarditis caused by *Helicobacter cinaedi*

**DOI:** 10.1186/s12879-014-0586-0

**Published:** 2014-11-18

**Authors:** Hanni Bartels, Daniel Goldenberger, Oliver Reuthebuch, Juerg Vosbeck, Maja Weisser, Reno Frei, Veronika Bättig

**Affiliations:** Division of Infectious Diseases and Hospital Epidemiology, University Hospital Basel, Petersgraben 4, Basel, 4031 Switzerland; Division of Clinical Microbiology, University Hospital Basel, Petersgraben 4, Basel, 4031 Switzerland; Clinic for Cardiac Surgery, University Hospital Basel, Spitalstrasse 21, Basel, 4031 Switzerland; Institute of Pathology, University Hospital Basel, Schönbeinstrasse 40, Basel, 4031 Switzerland

**Keywords:** Helicobacter cinaedi, Infective endocarditis, Broad-range bacterial PCR, Cytolethal distending toxin gene (ctd)

## Abstract

**Background:**

Up to 20% of all infective endocarditis are blood culture-negative and therefore a diagnostic challenge. Here we present the case of an infective endocarditis due to *Helicobacter cinaedi* finally diagnosed using different molecular methods. This highly fastidious gram-negative spiral rod is increasingly recognized as a human pathogen, above all in immunocompromised patients. So far *H. cinaedi* has been associated with bacteremia, cellulitis, arthritis and meningitis.

**Case presentation:**

A 71-year-old man presented with fever and progressive dyspnea for weeks. He was immunocompromised by long-term steroid therapy. As one major and two minor Duke’s criteria (vegetation, fever and aortic valve stenosis as predisposition) were present, an infective endocarditis was suspected and an empiric therapy with amoxicillin/clavulanic acid and gentamicin was established. The persistent severe aortic regurgitation resulted in a valve replacement. Histological evaluation of the aortic valve showed a polypous-ulcerative endocarditis. Gram stain and culture remained negative. Broad-range bacterial PCR targeting the 16S *rRNA* gene on the biopsy of the aortic valve identified *H. cinaedi* as the causative agent. The antibiotic therapy was simplified accordingly to ceftriaxone and gentamicin with a recommended duration of 6 weeks. Ten days after valve replacement the patient was discharged. To complete our molecular finding, we sequenced nearly the complete 16S *rRNA* gene (accession number KF914917) resulting in 99.9% identity with *H. cinaedi* reference sequences. Based on this result, 2 species-specific PCR tests amplifying part of the *ctd* gene were established and applied to the valve specimen. The 2 PCRs confirmed *H. cinaedi*. In addition, we analyzed stool, urine and saliva from the patient using *H. cinaedi* PCR. The fecal and urine specimen showed a positive signal, saliva was PCR-negative.

**Conclusion:**

We identified *H. cinaedi* as causative agent of a culture-negative endocarditis in an immunocompromised patient using broad-range and specific PCR. In addition to 2 cases from Japan presented on international meetings in 2010 and 2013, our case report shows that *H. cinaedi* should be recognized as additional causative organism of infective endocarditis. The use of molecular diagnostic techniques proved to be a powerful complement for the detection of blood culture-negative infective endocarditis.

**Electronic supplementary material:**

The online version of this article (doi:10.1186/s12879-014-0586-0) contains supplementary material, which is available to authorized users.

## Background

*Helicobacter cinaedi* is a spiral-shaped gram-negative rod, previously named *Campylobacter cinaedi* and was transferred to the genus *Helicobacter* in 1991 based on molecular phylogenetic analyses [1]. First detected from rectal swabs in homosexual men in 1984, *H. cinaedi* is considered an enterohepatic colonizer of the lower gastrointestinal tract of numerous mammals [2]. *H. cinaedi* is increasingly recognized as a human pathogen, and has been isolated from both immunocompetent [3] and from immunocompromised [4],[5] patients. So far, cases of bacteremia [5]-[7], gastroenteritis [8], cellulitis [3],[4], arthritis [9], meningitis [10], myopericarditis [11] and infections in asplenic patients have been reported [12]. *H. cinaedi* is extremely challenging to isolate, and therefore needs special laboratory practice.

The microaerophilic bacterium appears on agar plates as swarming thin film and should be preferably stained with acridine orange and not using Gram staining [13]. In a very recent study, Araoka H. et al. [6] showed that 45% of blood cultures showed growth with *H. cinaedi* only after >5 days of incubation.

## Case presentation

A 71-year-old man presented to the emergency room of a district hospital with progressive dyspnea. He reported experiencing fatigue, recurrent chills and fever spikes for several weeks. In his medical history was a polymyalgia rheumatica treated with long-term steroids (15 mg/d) and a moderate aortic stenosis. On admission, the patient was afebrile (37.2°C), tachycard (118/min.) and tachypnoeic (40/min.) with a blood pressure of 125/55 mmHg. There were bibasilar crackles on lung auscultation and cardiac examination revealed a 3/6 systolic and a 2/6 diastolic murmur. Laboratory analysis showed a leukocytosis of 21.2 G/l (range 3.5-10 G/l), an elevated C-reactive protein of 54 mg/l (<5 mg/l) and a creatinine level of 123 μmol/l (range 49–97 μmol/l). A pulmonary edema due to acute heart failure was diagnosed, and the patient had to be transferred to the intensive care unit for respiratory non-invasive ventilation. A transesophageal echocardiography (TEE) showed a thickened left coronary cusp with several mobile structures on its surface, together with a concomitant aortic regurgitation. There was no evidence of a perivalvular abscess. According to the modified Duke’s criteria [14] one major criterion (vegetation on the aortic valve) and two minor criteria (fever and aortic valve stenosis as a predisposition) were present, leading to the diagnosis of a possible infective endocarditis (IE). Therefore, an empiric intravenous antibiotic treatment with amoxicillin/clavulanic acid (2.2 g every 4 hours) and gentamicin (70 mg every 8 hours) was started. Subsequently, fever decreased but all blood cultures remained negative despite the fact that they had been taken before starting antibiotic therapy. Eight days after admission, the patient was transferred to our tertiary-care hospital for an urgent valve replacement because of deteriorating heart failure. Intra-operatively, a large aortic vegetation attached to the left coronary cusp and an abscess cavity with destruction of the cusp and aortic root were seen. A Mini-Root replacement with a Freestyle Aortic Root Heart Valve and an annuloplasty to repair the mitral valve was performed. Post-operatively the patient developed a third-degree atrio-ventricular block, necessitating the implantation of a pacemaker.

**Figure 1:**
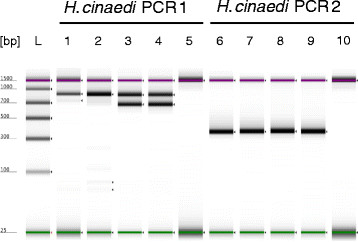
**Conventional 40-cycle** ***Helicobacter cinaedi*** **PCR 1 and 2 targeting the** ***cdt*** **gene resulting in 659-bp and 359-bp amplicons, respectively.** Polyacrylamid-based gel electrophoresis of PCR products using Agilent 2200 Tape Station (Agilent Technologies). L, molecular weight ladder; lane 1 and 6, 400 ng DNA of aortic valve (formalin-fixed); lane 2 and 7, 80 ng DNA of aortic valve (formalin-fixed); lane 3 and 8, 250 ng DNA of aortic valve (fresh); lane 4 and 9, 50 ng DNA of aortic valve (fresh); lane 5 and 10, negative control (5 μl of elution buffer).

Histology of the aortic valve showed a polypous-ulcerative endocarditis but Gram stain and conventional aerobic and anaerobic culture remained negative. Finally, broad-range bacterial PCR targeting the first half of the 16S *rRNA* gene on the fresh resected aortic valve identified *H. cinaedi* as the causative agent [15]. To complete our molecular finding, we amplified and sequenced nearly the complete 16S *rRNA* gene and submitted the 1458-bp-long sequence to the GenBank data base (accession no. KF914917). Comparison to *H. cinaedi* reference sequences resulted in a one-nucleotide difference (99.9% similarity) to reference strain PAGU0626 (accession no. AB275328) and 5 further *H. cinaedi* strains published in 2007 [3]. In addition, we established 2 species-specific PCR tests amplifying part of the cytolethal distending toxin gene (*ctd)* based on the study of Oyama K et al. [16] and applied them to the fresh as well as to the formalin-fixed paraffin-embedded (FFPE) valve specimen. Deparaffinization of the FFPE specimen was done using a xylol incubation step, then the fresh and dried FFPE sample were extracted with the QIAamp Mini Kit (Qiagen). The 2 independent PCRs including sequencing of the PCR amplicons confirmed the detection of *H. cinaedi* in both specimens (Figure 1). Sequence analysis of unspecific PCR products (approx. 850 bp) from the biopsy extracts of PCR 1 assay resulted in human DNA. The negative PCR result from the FFPE specimen in PCR 1 (expected fragment length 659 bp) could be explained by degradation or crosslinking of the DNA by formalin. Furthermore, we collected stool, urine and saliva from the patient 3 weeks after valve replacement and analyzed the 3 samples using nested *H. cinaedi* PCR combining the 2 PCR assays mentioned above according to [16]. The fecal and urine specimen showed a positive signal only after reamplification, saliva was PCR-negative.

Having confirmed *H. cinaedi* as the causative agent of IE, completion of treatment with amoxicillin 2 g every 4 hours was suggested. Unfortunately, the patient did not tolerate this therapy. Therefore, we had to change the antibiotic treatment to ceftriaxone 2 g in combination with gentamicin 180 mg every 24 hours. A treatment duration of six weeks after valve replacement was recommended. On day ten after valve replacement the patient was discharged in a rehabilitation clinic. Over time, the patient’s condition gradually improved and the inflammation parameter normalized. Five weeks after cardiac surgery and six weeks after start of antibiotic treatment, the patient discontinued intravenous antibiotic therapy against medical advice because of non-hazardous adverse events. In a follow up examination six weeks after cessation of therapy the patient presented still in good clinical condition with normal inflammation parameters. So far, no evidence for recurrence was found, however long term follow up is pending.

To our knowledge, this is the first published case of an infective endocarditis with *H. cinaedi*. Additional cases of IE associated with *H. cinaedi* have been described in two abstracts presented at international conferences [17],[18]. Taking into account these further cases, the clinical significance of *H. cinaedi* in invasive infections such as IE should be taken seriously. Moreover, *H. cinaedi* could be considered as an additional causative pathogen of IE, especially in cases of culture-negative IE.

Up to 20% of all IE remain blood-culture negative [19],[20] and are therefore still a diagnostic and therapeutic challenge. Beside antibiotic pre-treatment, one of the most common reasons for culture-negative IE are infections with obligate intracellular or fastidious organisms [19]. Due to the highly fastidious characteristics of *Helicobacter* species*,* independent molecular tests represent powerful alternatives to detect and identify these organisms. In the reported case, all blood cultures and the conventional cultures of the resected valve remained negative, even though the diagnosis of infective endocarditis was definitely demonstrated by histologic examination and intra-operatively described intra-cardiac abscess. Only broad-range bacterial PCR of the biopsy of the aortic valve and species-specific PCR of purified DNA from the histological specimen identified *H. cinaedi* as the causative agent. This case highlights the importance of alternative diagnostic approaches like PCR and sequence analysis (e.g. bacterial broad-range PCR targeting the 16S rRNA gene) for microbiological diagnosis, particularly for IE.

The detection of *H. cinaedi* DNA from stool and urine samples 3 weeks after initiating antimicrobial treatment is in congruency to the data of Oyama K. et al. [16], describing patients with fever and/or cellulitis. This findings may further indicate colonization of *H. cinaedi* in the enterohepatic tract.

A disadvantage of the molecular techniques is the lack of antimicrobial susceptibility testing. In the reported case, valve replacement and intravenous antibiotic treatment with amoxicillin/clavulanic acid followed by a combination of gentamicin and ceftriaxone, showed a successful clinical response and outcome. This antibiotic treatment is in accordance with previously published cases of invasive infections with *H. cinaedi*, where culture and therefore microbiologic resistance testing was available [4],[5]. An epidemiologic study of 23 patients with *H. cinaedi* associated illness by Kiehlbauch JA et al. [4] reported that treatment with a penicillin, tetracycline, or aminoglycoside may be more effective than treatment with cephalosporins, erythromycin, or ciprofloxacin. In particular, isolates can be resistant against quinolones. Nevertheless, scant data on treatment of infections with *H. cinaedi* has been published. The additional cases of IE due to *H. cinaedi* mentioned above were treated with a combined antibiotic therapy consisting of ampicillin/gentamicin [18] and ceftriaxone followed by levofloxacin [17]. There are no existing guidelines for choice or for duration of antibiotic treatment in cases of IE with *H. cinaedi*. Since recurrent infections are described [5], prolonged and combined antibiotic treatment is recommended.

## Conclusions

We identified *H. cinaedi* as a causative agent of a culture-negative endocarditis in an immunocompromised patient using broad-range bacterial (16S rRNA gene) and specific (*ctd* gene*)* PCR. In addition to two cases from Japan presented at international meetings in 2010 and 2013 [17],[18], our case report shows that *H. cinaedi* should be recognized as an additional causative organism of IE. The use of molecular techniques proved to be a powerful diagnostic adjunct for blood culture-negative IE.

### Consent

Written informed consent was obtained from the patient for publication of this Case report. A copy of the written consent is available for review by the Editor of this journal.

## Authors’ contributions

HB and VB drafted the manuscript and collected clinical data, DG carried out microbiological analysis including PCR and sequencing analysis and drafted part of the manuscript. RF and MW participated in coordination and design of the study and helped to draft the manuscript. OR provided clinical specimens and participated in the design of the study. JV carried out the histological analysis and participated in the design of the study. All authors read and approved the final manuscript.

## Authors’ original submitted files for images

Below are the links to the authors’ original submitted files for images. Authors’ original file for figure 1
