# Supplementary figures and images for: First case of infective endocarditis caused by Helicobacter cinaedi
Source: BMC Infect Dis. 2014 Nov 18;14:586. doi: 10.1186/s12879-014-0586-0 (PMC4243372; doi:10.1186/s12879-014-0586-0)

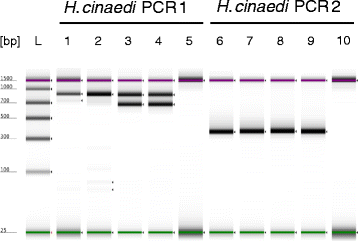

Supplement: Supplementary file 1 — Authors’ original file for figure 1 [file 12879_2014_586_MOESM1_ESM.gif]
